# Medical Items

**Published:** 1897-09

**Authors:** 


### MEDICAL ITEMS.

Dr. A. W. Stirling has removed his offices from the Electric Building to rooms 505-506 Grand Opera House.

---

One should not expectorate on open street-cars and expect to rate as a gentleman.—*T. C. M., Lancet-Clinic.*

---

Dr. Samuel S. Briggs, of Nashville, has been elected Associate Professor of Surgery in the Medical Department of the University of the South, Sewanee, Tenn.

---

Dr. Cornelius Kollock, of Cheraw, South Carolina, died August 16th, aged seventy-two years. Dr. Kollock was a highly esteemed practitioner and particularly distinguished for his contributions to abdominal surgery.

---

During the administration of ether the most alarming danger signals are sudden pallor of the face, dilatation of the pupils, and darkening of the blood. When the symptoms present themselves the anesthetic should at once be withdrawn, and resuscitating measures instituted.—*Hearn.*

---

Another new book is to be added to our rapidly increasing literature on pediatrics. A book entitled "About Children," is announced by the Medical Gazette Publishing Company, of Cleveland, Ohio. The author is Dr. Samuel W. Kelley, Professor of Diseases of Children in the Cleveland College of Physicians and Surgeons.

*New York Medical Journal* says the following application for erysipelas of the face is highly recommended: Carbolic acid, tincture of iodine, alcohol, 1 part each; oil of turpentine, 2 parts; glycerin, 3 parts. Mix. Paint the affected area with this liniment every two hours, and then cover the face with aseptic tarlatan.

---

The next meeting of the Mississippi Valley Medical Association will be held in Louisville, on October 5, 6, 7 and 8, 1897. All railroads will offer reduced rates. The president, Dr. Thos. Hunt Stucky, and the Chairman of the Committee of Arrangements, Dr. H. Horace Grant, promise that the meeting will be the most successful in the history of the Association.

---

All doctors are invited to attend the Ninth Annual Meeting of the Tri-State Medical Society of Alabama, Georgia and Tennessee, to be held in the Senate Chamber of the State Capitol, Nashville, Tenn., Tuesday, Wednesday and Thursday, October 12, 13 and 14, 1897. Reduced railroad rates to the Tennessee Centennial. Frank Trester Smith, M.D., Secretary, Chattanooga, Tenn. W. F. Westmoreland, M.D., President, Atlanta, Ga. W. D. Haggard, Jr., M.D., Chairman Committee of Arrangements, Nashville, Tenn.

---

A few days ago we visited the *Atlanta Retreat,* owned and conducted by Dr. C. C. Stockard, and we were pleased to find a well-appointed institution, and one conveniently adapted for the care and treatment of patients addicted to drug habits. Besides, there is a good operating-room for surgical work, and complete facilities for sterilization and asepsis. Physicians placing their surgical cases in this retreat retain absolute charge of the same, with the help of trained nurses. We take pleasure in commending the institution as highly worthy of professional encouragement.

The Journal of the Carroll County (Georgia) Medical Society (Vol. 1, No. 2) is before us, and we learn that it is a bi-monthly journal "devoted to the elevation of the medical profession." It contains two good papers on Puerperal Eclampsia, one on Gonorrhea, and one on Inguinal Hernia. Our brethren of the Carroll county society are to be congratulated upon the enterprise and professional spirit shown in this publication of their proceedings. The secretary of the society is Dr. J. F. Cole, and the editor of the *Journal* is Dr. S. T. Harris, both of Carrollton, Ga.

---

The *Georgia Journal of Medicine and Surgery* is the latest addition to our exchange list, the first number of which is just received. It is published in Savannah and is owned and edited by Drs. St. J. B. Graham, D. E. Dudley and W. E. Fitch. The first issue contains a number of interesting original papers and several judicious selections from current medical literature, and other medical items. It is well printed, and for an initial number is very creditable to its editors. We take pleasure in wishing the new journal all the success which its proprietors anticipate.

---

Of the original members of the American Medical Association only four of those present at its organization are still living. These are Dr. Nathan S. Davis, of Chicago, an ex-president; Dr. John B. Johnson, of St. Louis, an ex-vice-president; Dr. Alfred Stillé, of Philadelphia, an ex-president, and Dr. David F. Atwalter, of Springfield, Mass. These constitute the revered remnant of a once able and aggressive body of medical men whose purpose was to lay broadly and enduringly the foundation of medical science on American soil. They builded even better than they knew, and the present generation is in no wise tardy in a generous expression of its appreciation of their noble and successful work.—*Ex.*

